# Angelica Sinensis Polysaccharide-Based Nanoparticles for Liver-Targeted Delivery of Oridonin

**DOI:** 10.3390/molecules29030731

**Published:** 2024-02-05

**Authors:** Henglai Sun, Jijuan Nai, Biqi Deng, Zhen Zheng, Xuemei Chen, Chao Zhang, Huagang Sheng, Liqiao Zhu

**Affiliations:** 1 College of Pharmacy, Shandong University of Traditional Chinese Medicine, Jinan 250355, China; shl7995@163.com (H.S.); ne19863675828@163.com (J.N.); dbqdyx1@163.com (B.D.); zhengzhenzhen515@163.com (Z.Z.); hdva329@163.com (X.C.); tougaotcm@163.com (C.Z.); 2 Key Laboratory of Traditional Chinese Medicine Classical Theory, Ministry of Education, Shandong University of Traditional Chinese Medicine, Jinan 250355, China

**Keywords:** nano-carrier, Angelica sinensis polysaccharide, pH-sensitive, oridonin, antitumor, drug delivery

## Abstract

The present work aimed to study the feasibility of Angelica sinensis polysaccharide (ASP) as an instinctive liver targeting drug delivery carrier for oridonin (ORI) in the treatment of hepatocellular carcinoma (HCC). ASP was reacted with deoxycholic acid (DOCA) via an esterification reaction to form an ASP-DOCA conjugate. ORI-loaded ASP-DOCA nanoparticles (ORI/ASP-DOCA NPs) were prepared by the thin-film water method, and their size was about 195 nm in aqueous solution. ORI/ASP-DOCA NPs had a drug loading capacity of up to 9.2%. The release of ORI in ORI/ASP-DOCA NPs was pH-dependent, resulting in rapid decomposition and accelerated drug release at acidic pH. ORI/ASP-DOCA NPs significantly enhanced the accumulation of ORI in liver tumors through ASGPR-mediated endocytosis. In vitro results showed that ORI/ASP-DOCA NPs increased cell uptake and apoptosis in HepG2 cells, and in vivo results showed that ORI/ASP-DOCA NPs caused effective tumor suppression in H22 tumor-bearing mice compared with free ORI. In short, ORI/ASP-DOCA NPs might be a simple, feasible, safe and effective ORI nano-drug delivery system that could be used for the targeted delivery and treatment of liver tumors.

## 1. Introduction

Hepatocellular carcinoma (HCC) is a malignant cancer with high mortality and serious threat to human life and health [1]. Early HCC is usually surgically removed, but the initial symptoms of HCC are mild and develop rapidly, and the best treatment period for surgical treatment is often missed at the time of diagnosis [2]. In addition to surgical treatment, chemotherapy is still the most commonly used treatment for advanced liver cancer. However, most chemotherapy drugs are highly toxic and low specific, which can lead to systemic toxicity and serious side effects, and patients are prone to drug resistance [3,4]. Nano-targeted agents can optimize the biodistribution, pharmacokinetics and pharmacodynamics of single or multiple drugs, thereby increasing the therapeutic index, achieving targeted drug delivery, prolonging drug release time, reducing systemic toxicity and enhancing antitumor effects [5,6,7].

There are a variety of receptors expressed on the plasma membrane of hepatoma cells or tumor vascular cells. The asialoglycoprotein receptor (ASGPR) is the most widely known target receptor in hepatocytes, is highly expressed on the surface of hepatic parenchymal cells and has an innate binding affinity for a variety of molecules containing galactose and *N*-acetyl-galactosamine, which can degrade proteins through the endocytic-lysosomal pathway, increasing the distribution of liver-targeted drugs in the liver and reducing adverse reactions [8,9]. The plant polysaccharides rich in galactose have the characteristics of natural targeting, wide source, high biosafety, low cost and easy availability. Therefore, they have become a research hotspot in targeted preparation carriers [10,11].

In recent years, research using natural polysaccharides as targeted drug carriers has attracted wide attention. Angelica sinensis polysaccharide (ASP) is one of the main active components of Angelica sinensis (AS), which is widely used in traditional Chinese medicine. ASP-based nanoparticles (NPs) are of great significance in targeted drug delivery systems, which can target colon, lung, spleen, liver, mesenchymal stem cells, etc. [12,13]. Xu et al. [14] prepared colon-targeting NPs based on ASP with pH-and redox-responsiveness to specifically release the naturally active compound ginsenoside Rh2 in the colonic inflammatory site, which greatly alleviated ulcerative colitis (UC) symptoms and improved gut microbial homeostasis. ASP is rich in galactose and has a strong affinity for ASGPR, which is expected to become a new type of intrahepatic drug delivery carrier [15,16]. Liu et al. [17] prepared liver-targeting NPs based on ASP with hypoxic responsiveness to specifically release Curcumin (Cur) in the tumor site, which could significantly inhibit the proliferation of tumor tissue in tumor-bearing mice. Deoxycholic acid (DOCA), also known as 3α,12α-dihydroxy-5β-cholan-24-oic acid, is a natural substance which is often used for the hydrophobic modification of biomaterials due to its rigid and highly hydrophobic steroid skeleton [18,19]. Zhang et al. [20] synthesized ASP-DOCA NPs for the delivery of doxorubicin (DOX). The results of in vitro and in vivo experiments showed that DOX/ASP-DOCA NPs specifically targeted HepG2 tumors through ASGPR, improving the accumulation of DOX/ASP-DOCA NPs in tumors. Guo et al. [21] prepared ASP and GA dual-targeted NPs for Cur delivery. Compared with free Cur, the NPs had higher anti-hepatoma efficiency and targeting ability.

Oridonin (ORI), an ent-kaurene tetracyclic diterpenoid compound, is isolated from Chinese herb Rabdosia rubescens with various biological and pharmacological activities including antitumor, anti-microbial and anti-inflammatory effects. However, the clinical application of ORI is limited due to its low solubility and poor bioavailability. In order to overcome these shortcomings, many strategies have been explored such as structural modification, nanotechnology, liposomes, etc. [22].

**Figure 1:**
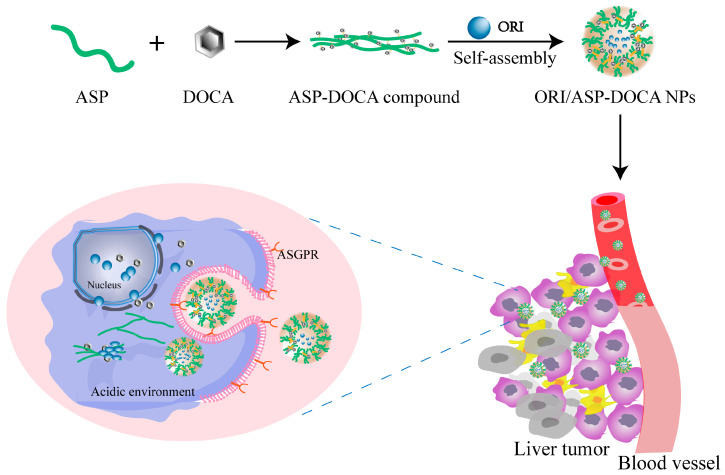
The design principle of ORI/ASP-DOCA NPs and its anti-liver tumor effect. Reprinted/adapted with permission from Ref. [23]. 2021, Dan Zheng.

The purpose of this study was to investigate the in vitro and in vivo anti-liver tumor effects of ORI/ASP-DOCA NPs. Firstly, crude ASP was prepared by water extraction and alcohol precipitation and purified by the freeze-thaw method, the dialysis method and gel chromatography to obtain purified ASP with a high galactose content. Secondly, ASP-DOCA was synthesized to prepare amphiphilic polymer carriers with pH response and liver targeting and then loaded with water-insoluble antitumor drug ORI to produce pH-sensitive ORI/ASP-DOCA NPs, as shown in Figure 1. The particle size, polydispersity index (PDI), zeta potential and morphology of ORI/ASP-DOCA NPs were characterized. The acid-triggered degradation and drug release behavior of ORI/ASP-DOCA were evaluated under different pH values. Thirdly, HepG2 and HeLa cells were used as models to evaluate the cytotoxicity, liver targeting and cell uptake of ORI/ASP-DOCA NPs in vitro. Finally, an H22 tumor-bearing mouse model was used to further investigate the in vivo liver targeting, safety and antitumor effects of ORI/ASP-DOCA NPs.

## 2. Results and Discussion

### 2.1. Characterization of ASP

**Figure 2:**
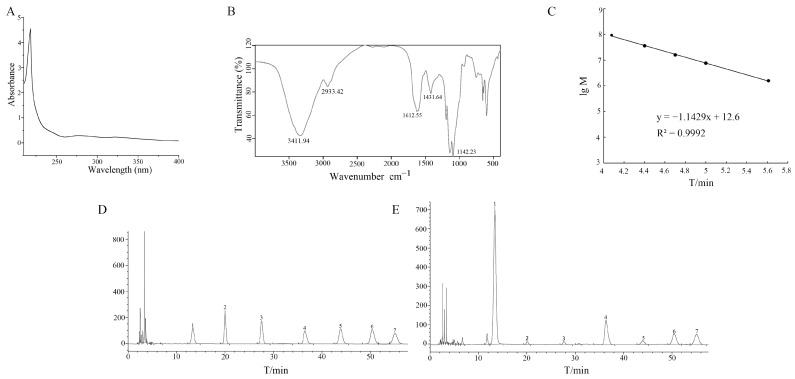
(**A**) UV absorption spectrum scanning results of ASP; (**B**) FT-IR spectrum of ASP; (**C**) Dextran corresponding molecular weight standard curve; (**D**) HPLC chromatogram of monosaccharide standard solution mixture; (**E**) HPLC chromatogram of ASP solution. (1: PMP; 2: d-mannose; 3: Rhamnose; 4: d-galacturonic acid; 5: d-glucose; 6: d-galactose; 7: Arabinose).

The purified ASP was white amorphous powder. The results of the phenol-sulfuric acid method showed that the content of polysaccharide in ASP was as high as 83%, and the UA method showed no absorption peaks at 260 nm and 280 nm (Figure 2A), indicating that there was no protein and nucleic acid and the purity of ASP was high. Fourier transform infrared spectroscopy (FT-IR) results showed that there was a broad peak at 3700–3100 cm^−1^, which was the typical O-H absorption peak in the polysaccharide sample. Absorption peaked at 3000–2800 cm^−1^, which was caused by C-H bond stretching vibration in the polysaccharide (Figure 2B).

The ASP solution was determined by high performance liquid chromatography (HPLC) and combined with the standard curve (Figure 2C). The retention time of the ASP solution was substituted into the standard curve to obtain the corresponding molecular weight of the elution peak, so the average molecular weight of ASP was about 114,025 Da.

In order to further determine the monosaccharide composition of ASP, ASP was detected by HPLC after acid hydrolysis. According to the retention time and HPLC analysis of the monosaccharide standard, it was concluded that ASP was composed of d-mannose, rhamnose, d-galacturonic acid, d-glucose, d-galactose and arabinose (Figure 2D,E) and that the content ratio of six monosaccharides was about 2.24:3.17:46.46:5.71:17.89:23.34. It could be seen that high concentrations of galactose in ASP might contribute to a good affinity for ASGPR [24].

### 2.2. Characterization of ASP-DOCA Compound

**Figure 3:**
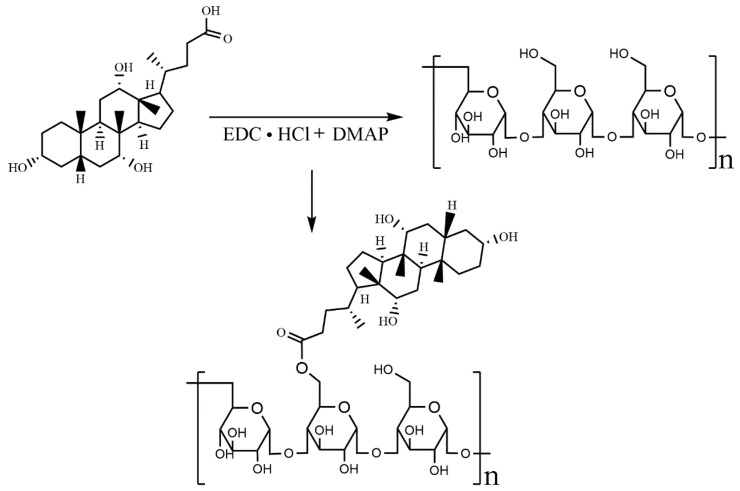
Synthetic route of the ASP-DOCA compound.

**Figure 4:**
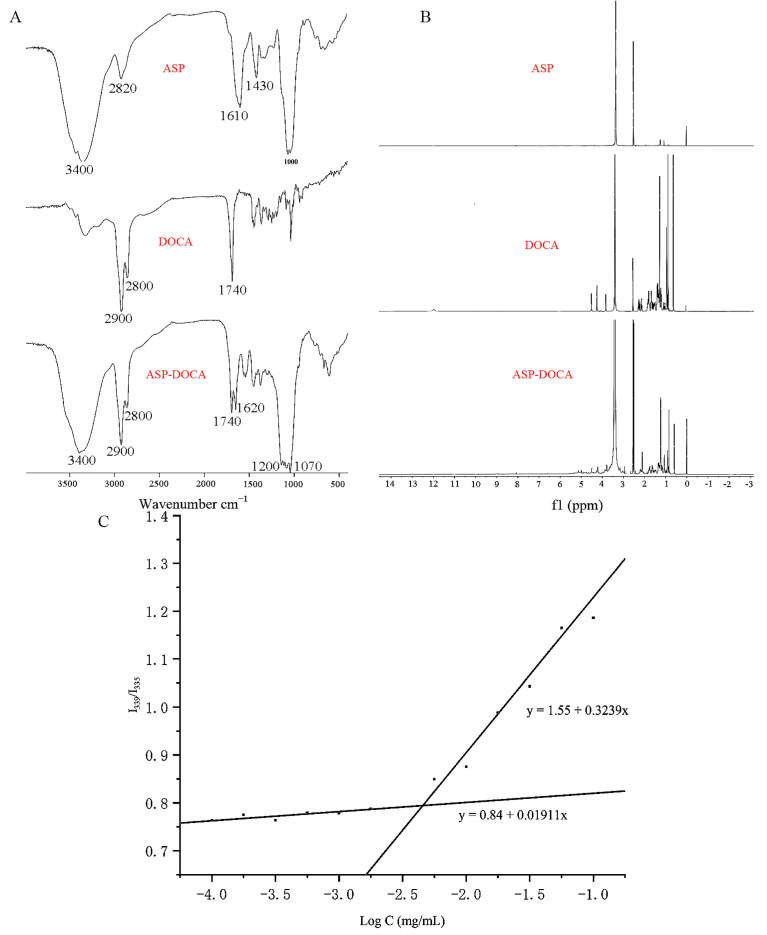
FT-IR (**A**) and ^1^H-NMR Spectra (**B**) of ASP, DOCA and the ASP-DOCA compound; (**C**) CAC of the ASP-DOCA compound.

The ASP-DOCA compound was synthesized by an esterification reaction using EDC·HCL and DMAP as reaction catalysts (Figure 3). The infrared spectrum showed the characteristic absorption peak of the ester. As shown in Figure 4A, the absorption peak at 1740–1620 cm^−1^ was attributed to ν_C = O_, which formed the ester. The result of ^1^H-NMR spectra was similar to that of FT-IR, which further verified the synthesis of the ASP-DOCA compound. As shown in Figure 4B, peaks ranging from 2.5 to 4.5 ppm attributed to ASP and the new peak appearing in the range of 0.5–2.5 ppm correspond to the hydrogen proton of DOCA, indicating the successful introduction of ASP into DOCA.

The ASP-DOCA compound could form NPs in an aqueous environment under probe ultrasonic treatment. In the presence of pyrene as a hydrophobic probe, the CAC of ASP-DOCA NPs in a water environment was measured by fluorescence analysis [25]. As shown in Figure 4C, the intensity ratio (peak I_1_/I_3_) of the pyrene emission spectrum began to decrease when ASP-DOCA NPs were formed. The linear graph was drawn with the logarithmic concentration and intensity ratio (peak I_1_/I_3_) of ASP-DOCA NPs, and the breakpoints of the two lines were measured as CAC values. The CAC of ASP-DOCA was calculated to be 0.0047 mg/mL, indicating that ASP-DOCA NPs easily formed amphiphilic NPs in an aqueous solution.

### 2.3. Synthesis and Characterization of ORI/ASP-DOCA NPs

**Figure 5:**
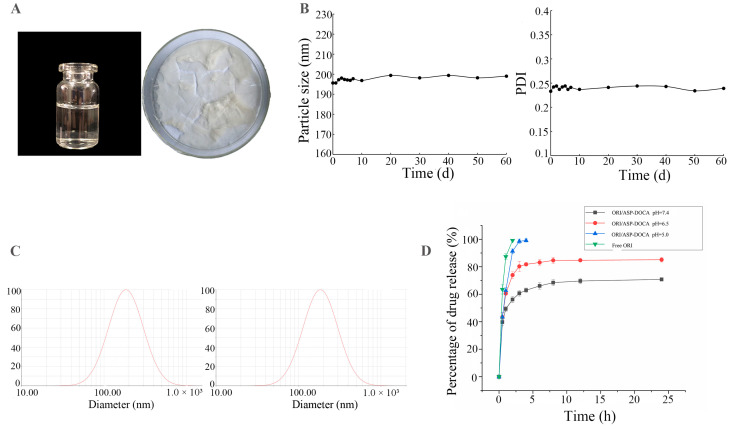
(**A**) ORI/ASP-DOCA NPs solution and its freeze-dried powder; (**B**) The relationship between the particle size and dispersion coefficient of ORI/ASP-DOCA NPs and storage time; (**C**) Particle size distribution of ORI/ASP-DOCA NPs; (**D**) The drug release curves of free ORI and ORI/ASP-DOCA NPs at different pH values.

A milk-white ORI/ASP-DOCA NPs solution was prepared by the thin-film water method, and it became a white spongy solid powder after freeze-drying (Figure 5A). The standard curve of ORI was *y* = 30.688*x* − 24.034 (*R*^2^ = 0.9997), and the linear relationship was good in the range of 2.006–200.06 μg/mL. The average EE and DL of ORI/ASP-DOCA NPs were (9.2 ± 1.21) % and (63.6 ± 0.24) %, respectively. As shown in Figure 5B, the particle size and PDI of ORI/ASP-DOCA NPs were slightly increased before and after storage, but the effect on the subsequent experiment was small, indicating that the prepared ORI/ASP-DOCA NPs were relatively stable. The comparison of the ASP-DOCA NPs and the ORI/ASP-DOCA NPs revealed different particle size, PDI and zeta potential characteristics. As shown in Figure 5C and Table 1, ORI/ASP-DOCA NPs exhibited a small particle size of 195 nm with a narrow size distribution (PDI = 0.233). The small particle size facilitated escape from the reticuloendothelial system and improved passive targeting performance in vivo by enhancing penetration and retention effects. Zeta potential, as an indicator of surface charge, can characterize the long-term stability of the particle system. The larger the absolute value of zeta potential, the higher the surface charge of NPs, and it is not easy to aggregate in the suspension system [26,27]. Monosaccharide composition analysis showed that ASP contained galacturonic acid, which indicated that ASP was an acidic polysaccharide. The negative zeta potential of ASP-DOCA NPs and ORI/ASP-DOCA NPs may be related to the carboxyl group in galacturonic acid. The repulsion between the negative charges could increase the stability of the nanodrug delivery system [28]. In addition, the negatively charged hydrophilic surface of the nanodrug delivery system could go unrecognized and be swallowed by macrophages, which increased the specific uptake of tumor cells [29,30].

In the in vitro drug release test (Figure 5D), about 85% of free ORI was released from the dialysis bag within 1 h and completely released within 2 h. The in vitro release curves of ORI/ASP-DOCA NPs at pH 7.4, pH 6.5 and pH 5.0 were consistent with the two-stage exponential kinetic model. The burst release of ORI was usually attributed to unencapsulated drugs. Subsequently, the release of ORI was slow and sustained, which might depend on the drug diffusion in the core of the NPs. The results also showed that compared with pH 7.4, the cumulative release of ORI from ORI/ASP-DOCA NPs was significantly higher under weak acidic conditions of pH 5.0 and 6.5. In conclusion, these results indicated that ORI/ASP-DOCA NPs had pH responsive release characteristics.

**Figure 6:**
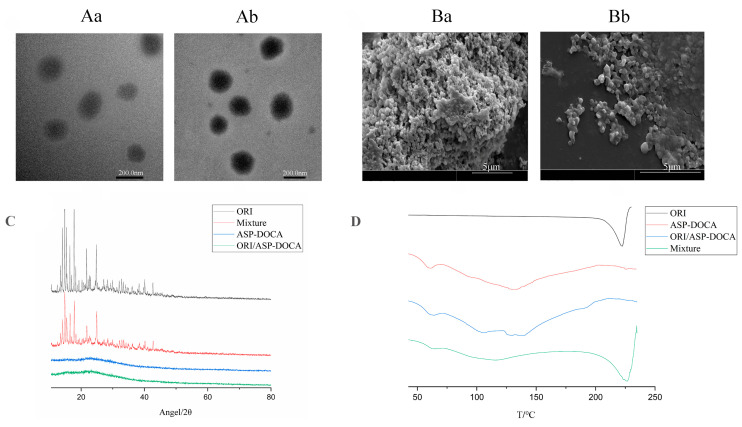
TEM images of ASP-DOCA NPs (**Aa**) and ORI/ASP-DOCA NPs (**Ab**); SEM images of ASP-DOCA NPs (**Ba**) and ORI/ASP-DOCA NPs (**Bb**); (**C**) XRD patterns of ORI, ORI/ASP-DOCA NPs, ASP-DOCA NPs, ORI and ASP-DOCA NPs physical mixture; (**D**) DSC spectrum of ORI, ORI/ASP-DOCA NPs, ASP-DOCA NPs, ORI and ASP-DOCA NPs physical mixture.

It could be seen from TEM and SEM images that ASP-DOCA NPs and ORI/ASP-DOCA NPs were nearly spherical, with particle sizes of 190 nm and 200 nm, respectively (Figure 6A,B). XRD analysis showed that there was no diffraction peak of ORI in ORI/ASP-DOCA NPs, which was different from ORI powder, ASP-DOCA NPs and their physical mixture (Figure 6C). These results indicated that ORI mainly existed in crystalline form in ORI powder and the physical mixture. The freeze-dried powder of ORI/ASP-DOCA NPs showed a flat curve without obvious diffraction peaks, indicating that ORI existed in an amorphous form in ORI/ASP-DOCA NPs.

DSC analysis was used to evaluate the melting behavior or crystallization of NPs, and the result was similar to that of XRD analysis (Figure 6D). Both the ORI powder and the physical mixture showed an acute exothermic peak at about 225 °C, indicating that ORI had crystallized. ORI/ASP-DOCA NPs did not show an endothermic peak at the same position, indicating that ORI existed in the ORI/ASP-DOCA NPs in an amorphous form. In short, XRD and DSC results showed that ORI was encapsulated in the core rather than adsorbed on the surface of ASP-DOCA NPs, which was preferable for a controlled release system.

### 2.4. Cytotoxicity of ORI/ASP-DOCA NPs In Vitro

**Figure 7:**
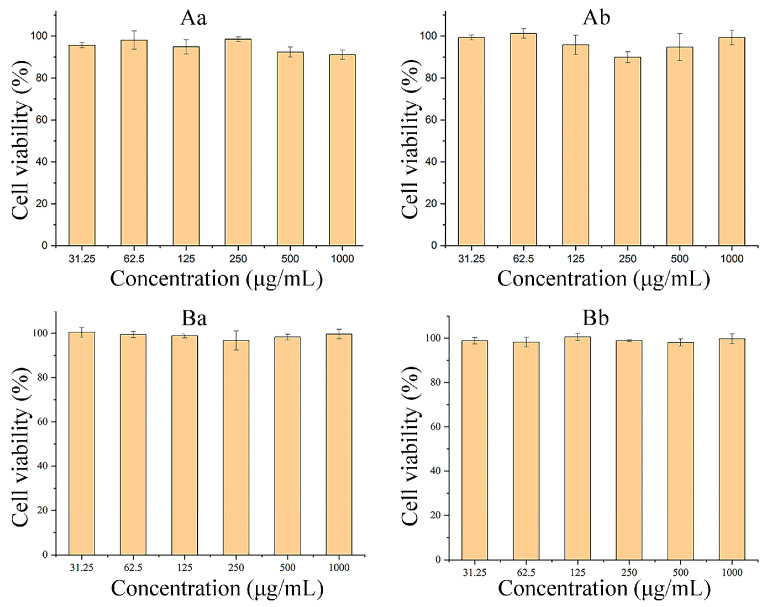
Cytotoxicity of ASP-DOCA NPs on HepG2 cells for 24 h (**Aa**) and 48 h (**Ab**); Cytotoxicity of ASP-DOCA NPs on HeLa cells for 24 h (**Ba**) and 48 h (**Bb**).

**Figure 8:**
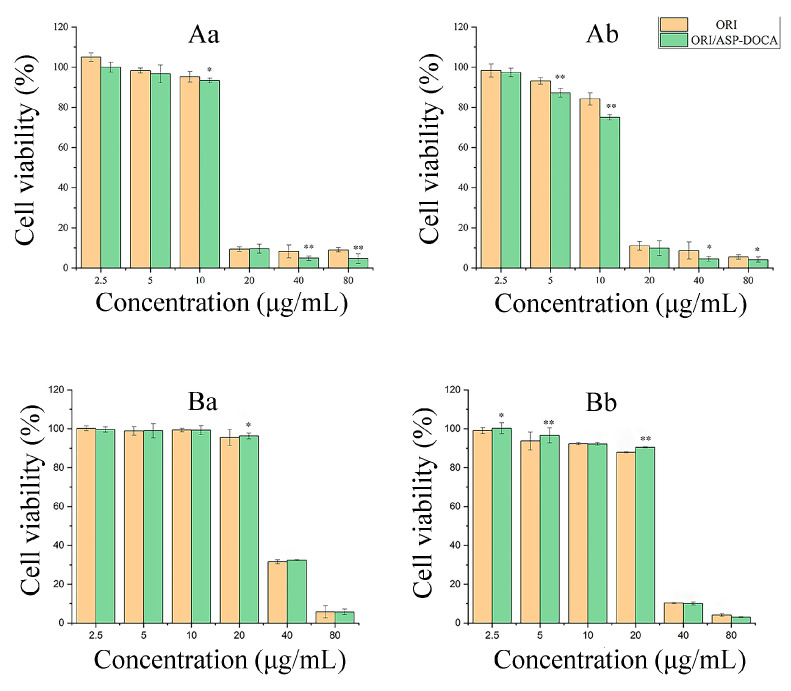
Cytotoxicity of free ORI and ORI/ASP DOCA NPs on HepG2 cells for 24 h (**Aa**) and 48 h (**Ab**); Cytotoxicity of free ORI and ORI/ASP DOCA NPs on HeLa cells for 24 h (**Ba**) and 48 h (**Bb**). (Note: compared with ORI group, * *p* < 0.05, ** *p* < 0.01).

The cytotoxicity of ASP-DOCA NPs on HepG2 and HeLa cells was respectively detected by MTT assay at 24 and 48 h. As shown in Figure 7, ASP-DOCA NPs at a concentration of 31.25–1000 μg/mL showed no significant cytotoxic effect on HepG2 (Figure 7A) and HeLa cells (Figure 7B), which indicated the security of ASP-DOCA NPs. However, ASP-DOCA NPs had significant cytotoxicity after the encapsulation of ORI. The results of free ORI and ORI/ASP-DOCA NPs on HepG2 cells showed that the cell viability decreased with the increase in drug concentration and time, indicating that the inhibitory effect of free ORI and ORI/ASP-DOCA NPs was dose- and time-dependent (Figure 8). In addition, there was a significant difference in the cytotoxicity of ORI/ASP-DOCA NPs compared with the free ORI group (Figure 8A). However, free ORI and ORI/ASP-DOCA NPs also had significant cytotoxicity to HeLa cells, but there was no significant difference between the two groups, indicating that ORI/ASP-DOCA NPs could specifically recognize HepG2 cells and show a higher toxicity in HepG2 cells than in HeLa cells (Figure 8B).

### 2.5. In Vitro Liver Targeting Study of ORI/ASP-DOCA NPs

**Figure 9:**
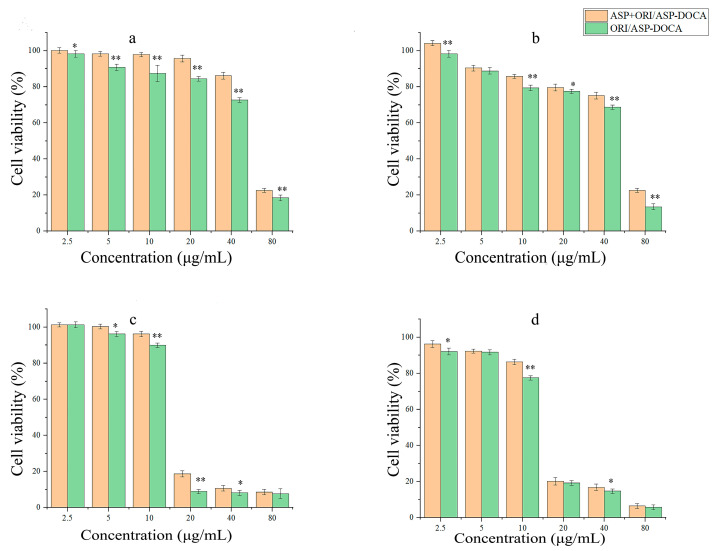
The in vitro targeting effect of ORI/ASP-DOCA NPs and ASP + ORI/ASP-DOCA NPs on HepG2 after 1 h (**a**), 2 h (**b**), 4 h (**c**) and 24 h (**d**). (Note: compared with ASP + ORI/ASP-DOCA NPs group, * *p* < 0.05, ** *p* < 0.01).

Furthermore, the MTT assay of HepG2 cells in the ORI/ASP-DOCA NPs group and the ASP + ORI/ASP DOCA NPs group were respectively carried out for 1, 2, 4 and 24 h, and the results are shown in Figure 9. At the same dosage and incubation time, the cell survival rate in the ASP + ORI/ASP-DOCA NPs group was higher than the normal group, because ASP could recognize and combine with ASGPR first and reduce the binding between ORI/ASP-DOCA NPs and ASGPR. Therefore, it was confirmed that ORI/ASP-DOCA NPs could specifically recognize ASGPR. With the increase in incubation time, the antagonistic effect of ASP was weakened, which was probably because ORI/ASP-DOCA NPs entered cells through other ways with the increase in incubation time [9,11,31].

### 2.6. Cellular Uptake of ORI/ASP-DOCA In Vitro

**Figure 10:**
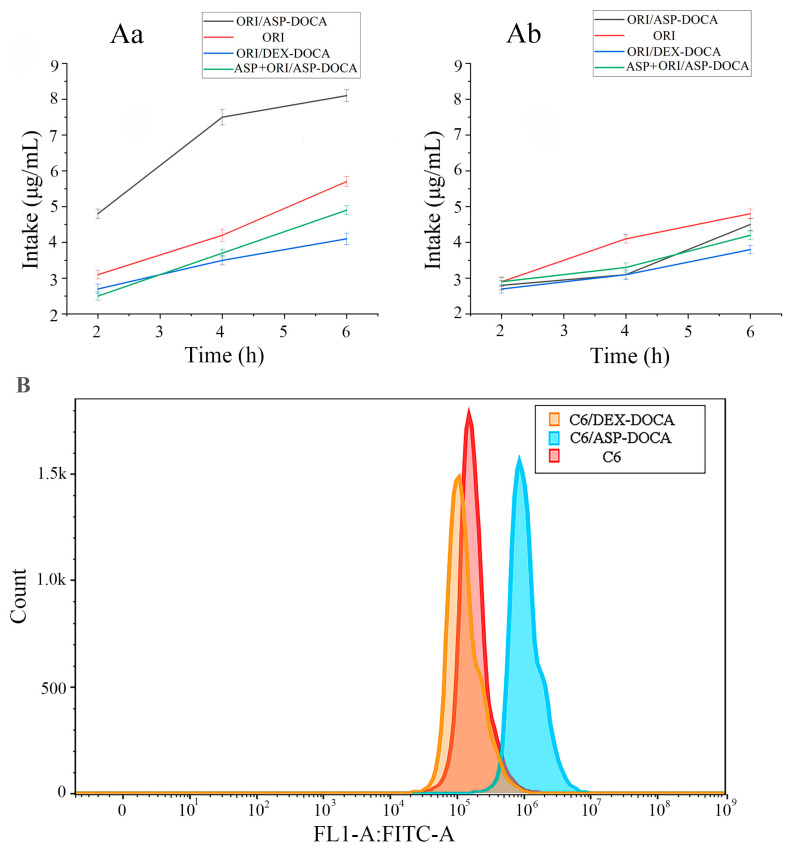
Cellular uptake of ORI, ORI/ASP-DOCA, ORI/DEX-DOCA and ASP + ORI/ASP-DOCA by HepG2 (**Aa**) and HeLa (**Ab**) cells; (**B**) Cellular uptake of C6, C6/ASP-DOCA and C6/DEX-DOCA NPs detected by flow cytometry.

The effective cellular uptake is crucial to evaluate the biological activities of chemotherapeutic formulations [32,33]. The cell-uptake status of ORI/ASP-DOCA NPs in vitro was observed by HPLC, CLSM and flow cytometry. As shown in Figure 10, Free ORI, ORI/ASP-DOCA and ORI/DEX-DOCA NPs significantly increased cell intake with the incubation time in a time-dependent manner. In addition, compared with the free ORI and ORI/DEX-DOCA groups, ORI/ASP-DOCA NPs could be absorbed more effectively by HepG2 cells, indicating that ORI/ASP-DOCA NPs enhanced the permeability of ORI to membranes. When HepG2 cells were incubated with ASP followed by ORI/ASP-DOCA NPs, the drug uptake in the ASP + ORI/ASP-DOCA group was lower than that in the ORI/ASP-DOCA NPs group, verifying that ORI/ASP-DOCA NPs could enter the cells through ASGPR-mediated endocytosis (Figure 10(Aa)). There was no significant difference in the cytotoxicity of HeLa cells between the ASP + ORI/ASP-DOCA group and the ORI/ASP-DOCA NPs group at the same incubation time, which also revealed that ORI/ASP-DOCA NPs were internalized into HepG2 cells by ASGPR receptor-mediated endocytosis (Figure 10(Ab)).

The results of drug uptake measurement by flow cytometry are shown in Figure 10B. The fluorescence intensity of C6/DEX-DOCA NPs was remarkably lower than that of free C6, which might be related to the fact that free C6 had a small molecular weight and could enter cells more quickly. The fluorescence intensity of cells in the C6/ASP-DOCA NPs group had been significantly enhanced, suggesting that ASP-DOCA NPs had an active targeting effect and could significantly enhance the drug concentration in liver tumor cells.

**Figure 11:**
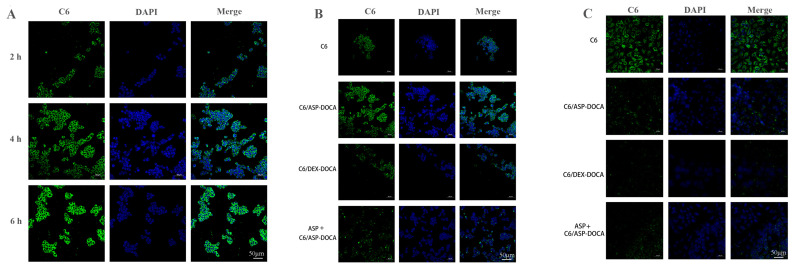
(**A**) CLSM images of HepG2 cells incubated with C6/ASP-DOCA NPs for different times of 2, 4, 6 h. (**B**) CLSM images of HepG2 cells incubated with different drugs for 4 h. (**C**) CLSM images of HeLa cells incubated with different drugs for 4 h.

The CLSM images of cell uptake are shown in Figure 11. The intracellular green fluorescence was significantly enhanced with lengthening incubation time, indicating that the uptake of C6/ASP-DOCA NPs by HepG2 cells was time-dependent (Figure 11A). The fluorescence intensity of the C6/ASP-DOCA NPs group was significantly stronger than that of the free C6 and C6/DEX-DOCA NPs groups (Figure 11B). However, the fluorescence intensity of the ASP-pretreated group was significantly weakened, indicating that C6/ASP-DOCA NPs could enter cells through ASGPR-mediated endocytosis. The fluorescence of the free C6 group was stronger than that of the C6/DEX-DOCA NPs and ASP + C6/ASP-DOCA NPs groups, which might be due to the small size of free C6, and free diffusion speed was faster than that of the NPs entering cells. Conversely, after the action on HeLa cells by different NPs, the fluorescence intensity of the free C6 group was stronger than that of other groups, and the fluorescence intensity of the C6/ASP-DOCA NPs group was significantly weakened, which was similar to that of the ASP + C6/ASP-DOCA NPs group and the C6/DEX-DOCA NPs group, indicating that C6/ASP-DOCA NPs had no targeting effect on HeLa cells (Figure 11C).

### 2.7. Liver Targeting and Antitumor Effect of ORI/ASP-DOCA NPs In Vivo

**Figure 12:**
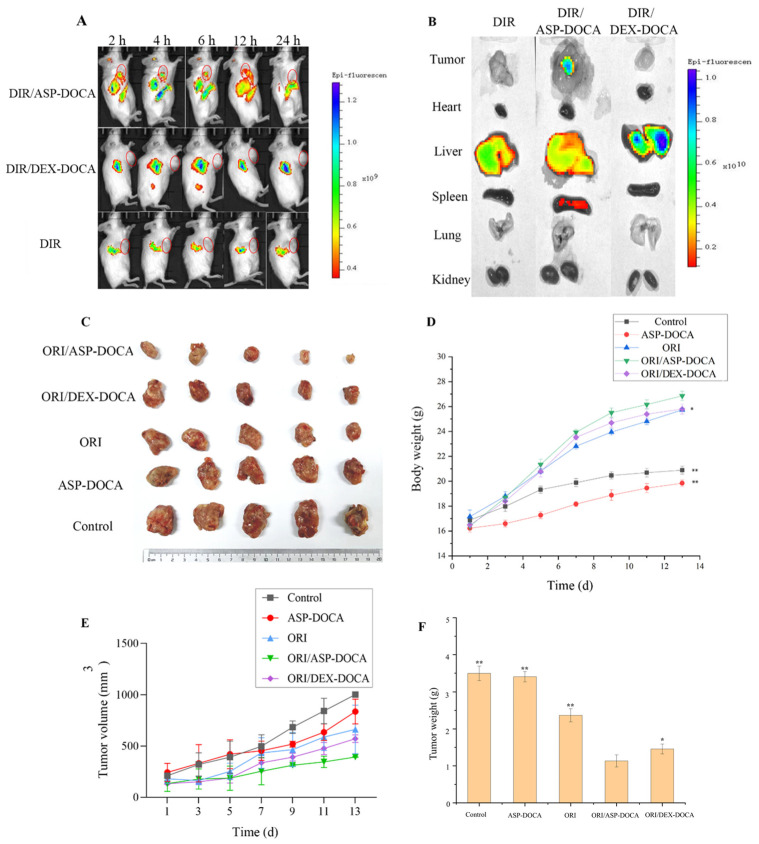
(**A**) Fluorescence images of DIR obtained by free DIR, DIR/ASP-DOCA and DIR/DEX-DOCA NPs at different time points of tumor-bearing mice in vivo; (**B**) DIR fluorescence imaging of the main isolated organs of tumor-bearing mice; (**C**) Tumor tissue images of tumor-bearing mice in each group; (**D**) Changes in the body weight of tumor-bearing mice in each group; (Note: compared with the ORI/ASP-DOCA NPs group, * *p* < 0.05, ** *p* < 0.01); (**E**) Changes in the tumor volume of tumor-bearing mice in each group; (**F**) Tumor tissue weight of tumor-bearing mice in each group (Note: compared with control group, * *p* < 0.05, ** *p* < 0.01).

The different intensity fluorescence signals were shown in the liver and tumor tissues of tumor-bearing mice after the intravenous injection of different DIR preparations through the tail vein. After 2 h of administration, the obvious fluorescence was observed in the tumor tissues (red circle-marking) of the DIR/ASP-DOCA group, and the fluorescence intensity was weakened gradually after 4–12 h of administration in the tumor (Figure 12A). After 24 h of administration, the fluorescence intensity in the tumor was weak, but the fluorescence intensity in the isolated tumor tissue and liver of mice was still strong, indicating that ORI/ASP-DOCA NPs was still not completely metabolized (Figure 12B). These results indicated that ORI/ASP-DOCA NPs exhibited good ASGPR receptor-mediated tumor targeting ability in H22 tumor-bearing mice, which was helpful to effectively improve the therapeutic effect.

The antitumor activity of different ORI preparations was evaluated by examining the tumor volume and weight of tumor-bearing mice. Compared with the free ORI group and the ASP-DOCA NPs group, the ORI/ASP-DOCA NPs and ORI/DEX-DOCA NPs groups could significantly inhibit tumor growth, and the antitumor effect of ORI/ASP-DOCA NPs was more obvious (Figure 12C,E). The tumor inhibition rates of the ORI/ASP-DOCA NPs and ORI/DEX-DOCA NPs groups reached 67.54% and 58.33%, respectively, which indicated that ORI/ASP-DOCA NPs could effectively reach the tumor site (Figure 12F). The above results were consistent with the results of in vivo imaging.

**Figure 13:**
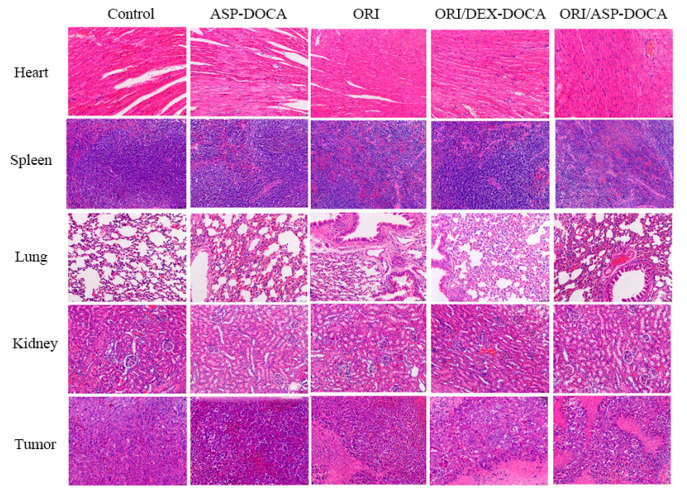
H & E staining on sections of hearts, spleens, lungs, kidneys and tumors in tumor-bearing mice in each group (200×).

The change in the body weight of tumor-bearing mice can indirectly reflect the safety of ORI/DEX-DOCA NPs in vivo. As shown in Figure 12D, compared with the free ORI group and the control group, the body weight of the ORI/ASP-DOCA group was significantly increased (*p* < 0.05), which indicated that ORI/ASP-DOCA NPs were safe for mice and could reduce the systemic toxicity of free ORI. In addition, the histological changes of the main organs of tumor-bearing mice were observed by H & E staining (Figure 13). There were no obvious signs of cell death in the hearts, spleens, lungs and kidneys of each treatment group, indicating that ORI/ASP-DOCA NPs had good safety. From the results of tumor tissue staining in mice, it can also be seen that the tumor cells in the ORI/ASP-DOCA NPs group had obvious apoptosis, which was the reason for inhibiting the growth of tumor tissues [34,35]. In summary, these results indicated that ORI/ASP-DOCA NPs were safe and effective in tumor-bearing mice.

**Figure 14:**
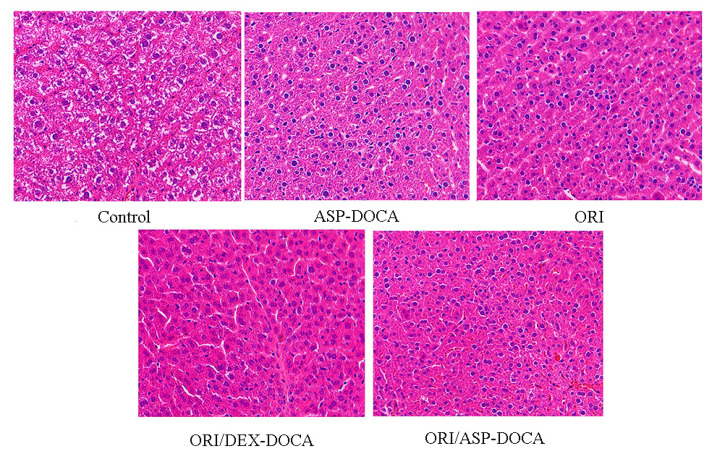
H & E staining of mouse liver sections in each group (200×).

The results of the H & E staining of the liver sections of tumor-bearing mice in each group are shown in Figure 14. In control group, the liver cells were disordered, the nucleus was abnormally enlarged, and there were different degrees of deformation, which may be related to the invasion and metastasis of liver cancer cells [36,37]. In the free ORI, ASP-DOCA NPs, ORI/ASP-DOCA NPs and ORI/DEX-DOCA NPs groups, the changes in liver cells in tumor-bearing mice were inhibited to varying degrees. Among them, the cells in the ORI/ASP-DOCA NPs group were closely arranged, the size was uniform, and the nucleus was slightly enlarged, indicating that the ORI/ASP-DOCA NPs could inhibit the invasion and metastasis of liver cancer cells.

## 3. Materials and Methods

### 3.1. Materials

The dry roots of *Angelica sinensis* (Oliv.) Diels were obtained from Zhonghe Chinese Medicine Pieces Co., Ltd. (Jinan, China). Anhydrous dimethyl sulfoxide (DMSO) and 3-(4,5-dimethylthiazol-2-yl)-2,5-diphenyltetrazolium bromide (MTT) were purchased from Sigma (St. Louis, MO, USA). d-Xylose, rhamnose (Rha), d-mannose (Man), d-galactose (Gal), l-arabinose (Ara), Deoxycholic acid (DOCA), 4-dimethylaminopyridine (DMAP), potassium bromide, DMSO-*d*_6_, Coumarin-6 (C6) and 1,1′-Dioctadecyl-3,3,3′,3′-tetramethylindotricarbocyanine iodide (DIR) were purchased from Yuanye Biotechnology Co., Ltd. (Shanghai, China). *N*, *N*-Dimethylformamide was purchased from Sinopharm Group Chemical Reagent Co., Ltd. (Shanghai, China). 1-ethyl-3-(3-dimethylaminopropyl) carbodiimide hydrochloride (EDC·HCl) was purchased from Macklin Biochemical Technology Co., Ltd. (Shanghai, China). ORI (98%) was purchased from Langze Biotech. Co. Ltd. (Nanjing, China). Standard d-mannose, Rhamnose, d-galacturonic acid, d-glucose, d-galactose and Arabinose were purchased from Yuanye Biotechnology Co., Ltd. (Shanghai, China). Dextran standards: T-410, T-50, T-25, T-15 and T-12 with molecular weight ranging from 410 to 12 kDa were purchased from Macklin Biochemical Co., Ltd. (Shanghai, China). DMEM medium and Trypsin EDTA (0.25%) were purchased from Gibco (Grand Island, NY, USA). Foetal bovine serum (FBS) was purchased from Tianhang Biotechnology Co., Ltd. (Zhejiang, China). RIPA cell lysate was purchased from Biyuntian Biotechnology Co., Ltd. (Shanghai, China).

Female BABL/c tumor-bearing mice, weighing 18 ± 2 g was provided by Weitonglihua Experimental Animal Technology Co., Ltd. (Beijing, China). All animals were housed in the Experimental Center of Shandong University of TCM (Jinan, China) for one week to applicability feeding, with free access to food and water.

### 3.2. Preparation and Physicochemical Characteristics of ASP

The crude ASP was prepared by water extraction and alcohol precipitation [38]. The dried roots of 200 g AS were soaked in 80% ethanol for 24 h and then boiled in distilled water to extract for 2 h. The extract solution was concentrated, and calcium hydroxide suspension was added until obvious flocculent precipitate appeared. The obtained solution was centrifuged at 4000× *g* rpm for 15 min to collect the supernatant, and sulfuric acid was added to adjust the pH value to 5.0–6.0. After centrifugation again, an equal volume of anhydrous ethanol was added to the precipitate for 24 h. The precipitate was centrifuged to obtain crude polysaccharide. Subsequently, proteins, pigments and monosaccharides were removed by the freeze-thaw method and the dialysis method (3.5 kDa, Shanghai yuanye Bio-Technology Co., Ltd., Shanghai, China), and then the crude polysaccharide was dissolved in distilled water and further purified using a Sephadex G-50 (Shanghai Yuanye Bio-Technology Co., Ltd., Shanghai, China). The purified fraction was combined, concentrated and lyophilized for further study.

The infrared spectrum and maximum ultraviolet absorption wavelength ASP were determined by FT-IR (Bruker Optics, Ettlingen, Germany) and ultraviolet spectrophotometer (UV, Hitachi, Tokyo, Japan), respectively. Monosaccharide composition was measured by reversed-phase HPLC after pre-column derivatization [39]. Sugar identification was achieved by comparison with reference sugars (d-glucose, d-xylose, d-mannose, arabinose, d-galactose, d-glucuronic acid and d-galacturonic acid), and the calculation of the molar ratio of the monosaccharide was carried out on the basis of the peak area of the monosaccharide.

HPLC conditions: injection volume 20 μL; column temperature: 30 °C; flow rate: 1.0 mL/min; detecting wavelength: 250 nm, mobile phase: acetonitrile-phosphate buffer (17:83, *v*/*v*).

Molecular weight for ASP was determined by high performance gel permeation chromatography (HPGPC, Agilent 1200, Santa Clara, CA, USA) [40]. Dextran standard and ASP were dissolved in 0.5 M Na_2_SO_4_ at a concentration of 1.0 mg/mL. The molecular weight of ASP was calculated by constructing a dextran standard curve, in which the logarithm of the molecular weight (lg M) of dextran standards ranging from 12,000 to 410,000 Da and the corresponding retention time were plotted as coordinates.

Agilent HPLC 1200 conditions: PL aquagel-OH MIXED-M (300 mm × 7.5 mm, 8 μm); mobile phase: 0.05 M Na_2_SO_4_ solution; flow rate: 1.0 mL/min; column temperature: 35 °C; detector: differential refractive detector; injection volume: 30 μL.

### 3.3. Synthesis and Characterization of ASP-DOCA Compound

Anhydrous DMSO (10 mL), DOCA (208.45 mg), EDC·HCL (76.55 mg) and DMAP (49.85 mg) were added into the conical flask and stirred at room temperature for 2 h, and the activated DOCA/DMSO solution was added dropwise to the ASP/DMSO solution [41]. The reaction was stirred at 45 °C for 72 h, and the insoluble matter was removed by filtration after the reaction was over. The filtrate was placed in the dialysis bag for 72 h, and the dialysate was replaced every 1 h. The dialysate was drained and filtered, and the white solid obtained was washed with methylene chloride and freeze-dried. Subsequently, the structure of ASP-DOCA compound was verified by FT-IR and ^1^H-NMR (BRUKER, Ettlingen, Germany). At the same time, the DEX-DOCA (nontarget) compound was synthesized by the above method due to subsequent control experiments.

The critical association concentration (CAC) of the ASP-DOCA compound was determined by the fluorescence method using pyrene as a hydrophobic fluorescent probe [42]. In short, the acetone solution of pyrene was added to a series of volumetric flasks. After the acetone was dried with nitrogen to form a pyrene film, different concentrations of the ASP-UDCA conjugate solution were added to the volumetric flask to make the final concentration of pyrene 6 × 10^−7^ M. The mixture of pyrene and the ASP-DOCA compound was placed in a constant temperature oscillating water bath (SHZ-A, Shanghai Boxun Medical Biological Instrument Co., Ltd., Shanghai, China) and balanced overnight at 37 °C. The emission wavelength was set to 372 nm, and the fluorescence intensity of the mixed solution at 339 nm and 336 nm was measured and recorded by scanning the 300–600 nm range with a F-7000 fluorescence spectrometer (Hitachi, Tokyo, Japan).

### 3.4. Establishment of Methodology of HPLC Analysis

HPLC analysis was performed on an Agilent 1260 (Agilent, Santa Clara, CA, USA). Ultraviolet detection was performed at 239 nm using the following parameters: Inertsil ODS-3 column (4.6 × 250 mm, 5 μm); flow rate of 1.0 mL/min; column temperature of 30 °C; injection volume of 20 μL. The mobile phase consisted of 60% acetonitrile and 40% water.

Standard curve: A total of 1.003 mg/mL of ORI was dissolved in methanol and then diluted to 2.006, 10.03, 20.06, 50.15, 100.3 and 200.6 μg/mL drug solution, separately. The above different concentrations of solution were injected into the chromatograph, and the standard curve was calculated according to the peak area (Y) and ORI concentration (X). In order to test the accuracy of the method, 2.006, 20.06 and 100.3 μg/mL were analyzed by HPLC (*n* = 6). Precision tests were performed on the above three samples twice a day for three days (*n* = 6).

### 3.5. Preparation and Characterization of ORI/ASP-DOCA NPs

ORI/ASP-DOCA NPs were prepared by the thin film water method [43]. ORI and ASP-DOCA were weighed and dissolved in DMF. The DMF was evaporated under reduced pressure at 80 °C until a uniform film was formed. Then, pure water was slowly added to it, and an ORI/ASP-DOCA NPs solution was obtained by ultrasonication for 15 min. The above solution was filtered through a microporous membrane (0.45 μm) and freeze-dried to obtain solid ORI/ASP-DOCA NPs.

The particle size, PDI and zeta potential of ASP-DOCA NPs and ORI/ASP-DOCA NPs were determined by laser particle size analyzer. The morphological images of ORI/ASP-DOCA NPs were obtained on a H-7650 transmission electron microscope (TEM, Hitachi, Tokyo, Japan) and scanning electron microscope (SEM, FEI Quanta, Hillsboro, OR, USA) to confirm their particle size and uniformity. The sample solution containing 0.1% (*w*/*v*) ORI/ASP-DOCA NPs was dropped on the copper grid for TEM observation. The excess sample was removed under the action of a filter, and the solution on the grid was allowed to dry naturally.

An X-ray diffractometer (XRD, Rigaku Ultima IV, Tokyo, Japan) and Q2000 differential scanning calorimeter (DSC, TA Instruments, New Castle, DE, USA) were used to study the state of ORI in ASP-DOCA NPs. XRD was performed using Cu Kα radiation at a scan rate of 5°/min operated at 40 kV and 100 mA. DSC analyses were performed with heating and cooling at a rate of 10 °C/min, and the empty aluminum sheet was used as a blank control.

The drug loading (DL) and encapsulation efficiency (EE) of ORI/ASP-DOCA NPs were determined. In short, the ORI/ASP-DOCA NPs solution was centrifuged at 15,000 rpm for 30 min, and the content of ORI was determined by HPLC, denoted as C_1_. The ORI/ASP-DOCA NPs solution was taken and placed in a bottle with methanol diluted to the scale. The total ORI content in the ORI/ASP-DOCA NPs solution was determined, denoted as total C_2_. The EE and DL of ORI in the NPs were calculated according to the following formula [44]:

$$\mathrm{EE}\;\left(\%\right)=\frac{C_{2}-C_{1}}{C_{2}}\;\times \;100\%$$

$$\mathrm{DL}\;\left(\%\right)=\frac{C_{2}-C_{1}}{C_{\mathrm{ORI}/\mathrm{ASP}-\mathrm{DOCA}}}\;\times \;100\%$$

### 3.6. In Vitro Drug Release

The in vitro drug release of ORI/ASP-DOCA NPs was evaluated by the dialysis method [45]. ORI and ORI/ASP-DOCA NPs (ORI concentration of 1 mg/mL) were first placed in a dialysis bag (3.5 kDa, Shanghai Yuanye Bio-Technology Co., Ltd., Shanghai, China) and then placed in 50 mL of phosphate buffer PBS (pH = 7.4, 6.5, 5.0), which was used as a release medium to simulate the in vivo environment. The oscillation condition was set at 37 °C and 120 r/min.

At different time intervals (0.5, 1, 2, 3, 4, 6, 8, 12, 24 h), 2 mL of medium was withdrawn while supplemented with an isothermal equal volume of dissolution medium. The sample solutions with ORI and ORI/ASP-DOCA NPs were filtered by 0.45 μm micropore film. The concentrations of ORI and ORI/ASP-DOCA NPs in the collections at different time points were determined by the above HPLC chromatographic conditions.

### 3.7. Cell Culture

Human HCC cells (HepG2, ASGPR receptor tumor cells) and human cervical cancer cells (HeLa) were obtained from Bomente Biology Science and Technology Co., Ltd. (Jinan, China). The cells were cultured in DMEM medium with 10% FBS and 1% penicillin/streptomycin in a 5% CO_2_ atmosphere incubator at 37 °C.

### 3.8. Cytotoxicity Assay

HepG2 and HeLa cells were used to investigate the cytotoxicity of ASP-DOCA NPs and ORI/ASP-DOCA NPs. HepG2 and HeLa cells were seeded in 96-well plates at a density of 1 × 10^4^ cells/well and incubated in a constant temperature incubator at 37 °C with 5% CO_2_ content. After 24 h and 48 h, the medium was discarded, and ORI, ASP-DOCA NPs and ORI/ASP-DOCA NPs with different mass concentrations (ORI mass concentrations were 2.5, 5, 10, 20, 40, 80 μg/mL) and four multiple pores were set for each concentration. Finally, MTT was added to each well, and cell viability was determined by using a microplate reader (Thermo Fisher, Waltham, MA, USA) at 490 nm. Cell viability was obtained through the following formula:

$$\mathrm{Cell}\;\mathrm{viability}\;\left(\%\right)=\frac{\mathrm{Drug}\;\mathrm{OD}\;-\;\mathrm{Blank}\;\mathrm{OD}}{\mathrm{Control}\;\mathrm{OD}\;-\mathrm{Blank}\;\mathrm{OD}}\;\times \;100\%$$

### 3.9. In Vitro Cellular Uptake Assay

#### 3.9.1. ORI Cellular Uptake Observed by HPLC

The digested HepG2 and HeLa cells were seeded in 96-well plates at a density of 4 × 10^5^ cells/well. The medium was added to each well and incubated at 37 °C and 5% CO_2_ for 24 h. Then, HepG2 and HeLa cells were treated with ORI, ORI/ASP-DOCA NPs, ASP + ORI/ASP-DOCA NPs and ORI/DEX-DOCA NPs 2, 4 and 6 h, respectively. The drug-containing medium was discarded, and cold RIPA was added to lyse the cells for 30 min. The cell fragments were scraped off and transferred to a centrifuge tube and centrifuged at 15,000 rpm for 10 min at 4 °C, and then supernatant was taken and transferred to a new centrifuge tube. The equal volume of cell lysis buffer and methanol solution were weighed, vortexed for 30 s and centrifuged again for 10 min at 15,000 rpm at 4 °C and analyzed by HPLC.

#### 3.9.2. Cellular Uptake Observed by Confocal Laser Scanning Microscope (CLSM)

Using fluorescent probe C6 as a model drug, the uptake of NPs in cells was observed by CLSM. HepG2 and HeLa cells were treated with the same protocol as HPLC analysis: firstly, HepG2 and HeLa cells were treated with free C6, C6/ASP-DOCA NPs, C6/DEX-DOCA NPs and ASP + C6/ASP-DOCA NPs. After incubation for 2, 4 and 6 h, the cells were washed with cold PBS three times and then fixed with cold paraformaldehyde (4%) for 15 min. The mixture was washed again with cold PBS three times (3–5 min each time), and a small amount of DAPI solution was added for 5 min to stain the nucleus again. The staining solution was discarded, and the stained cells were washed with cold PBS for 3–5 min each time to remove free DAPI. The cells were observed by laser confocal microscope (Zeiss, Jena, Germany) at excitation and emission wavelengths of 456/504 nm and 364/454 nm, respectively, and the images were recorded.

#### 3.9.3. Cellular Uptake Observed by Flow Cytometry

HepG2 cells were treated with the same protocol as confocal analysis. After 2 h of culture, the samples were washed with PBS buffer three times, digested and centrifuged, the appropriate amount of PBS was added for re-suspension, and the fluorescence intensity was measured by flow cytometry (Beckman, Brea, CA, USA) at 456/504 nm.

### 3.10. In Vitro Liver Targeting Study of ORI/ASP-DOCA NPs

HepG2 cells were incubated with ASP, and the ASGPR on HepG2 cells was combined with ASP and then treated with ORI/ASP-DOCA NPs. HepG2 and HeLa cells were digested into a single cell suspension and inoculated into 96-well plates at a density of 1 × 10^4^ cells/well. They were incubated at 37 °C and 5% CO_2_ for 24 h and setting the ORI/ASP-DOCA NPs group and ASP + ORI/ASP-DOCA NPs group. The ORI/ASP-DOCA NPs group was not treated, while the ASP + ORI/ASP-DOCA NPs group was treated with ASP for 2 h before ORI/ASP-DOCA NPs were added. After incubation for 1, 2, 4 and 24 h, the OD value was measured using microplate reader at 490 nm.

### 3.11. In Vivo Liver Targeting and Antitumor Analysis

To inquire whether ASP-DOCA NPs had liver tumor targeting and antitumor efficacy in vivo, H22 tumor-bearing mice models of liver tumor were established. When the tumor volume reached 90–120 mm^3^, the tumor-bearing mice were randomly divided into three groups (*n* = 5): the free DIR group, the DIR/ASP-DOCA NPs group and the DIR/DEX-DOCA NPs group. The mice were administered with 200 μg/kg dose by tail vein injection at 2, 4, 6, 12, 24 h, and then the fluorescence distribution images of tumor-bearing mice were collected using a small animal in vivo imaging system (IVIS Lumina XRMS). During the imaging process, the tumor-bearing mice were anesthetized by an isoflurane gas anesthesia system.

In addition, in order to further explore the in vivo antitumor effect and security of ORI/ASP-DOCA NPs, the tumor-bearing mice were also randomly divided into five groups (*n* = 5): the control group (saline injection), the free ORI group, the targeted group (ORI/ASP-DOCA group), the non-targeted group (ORI/DEX-DOCA group) and the carrier group (ASP-DOCA group). The ORI solution (20 mg/kg) was injected into the free ORI group. The ORI/ASP-DOCA NPs solution (20 mg/kg) was injected into the targeted group. The ORI/DEX-DOCA NPs solution (20 mg/kg) was injected into the non-targeted group. The ASP-DOCA NPs solution (100 mg/kg) was injected into the vehicle group. The drug was administered via tail vein injection once every three days and weighed once every two days. General characteristics such as the hair and mental state of the mice were observed every day.

After 14 days, the mice were sacrificed, and the tumors, livers, hearts, spleens, lungs and kidneys were separated from the body and weighed. Then, these tissues and organs were fixed overnight with 4% paraformaldehyde, paraffin-embedded sections, hematoxylin and eosin (H & E) staining and photographed. The tumor inhibition rate was calculated according to the following formula:

$$\mathrm{Inhibition}\;\mathrm{rate}\;(\%)=\frac{m_{1}-m_{2}}{m_{1}}\times 100\%$$

m_1_: Tumor weight in control group; m_2_: Tumor weight in administration group.

### 3.12. Statistical Analysis

All results are presented as the means ± standard deviation (SD). Statistical significance was analyzed using one-way ANOVA followed by *t*-test. Differences between groups were considered significant, where * *p* < 0.05, ** *p* < 0.01 were considered highly significant.

## 4. Conclusions

In the current study, we prepared ORI/ASP-DOCA NPs with excellent drug loading and sustained release properties and evaluated the antitumor potential of ORI/ASP-DOCA NPs through in vitro and in vivo studies. ORI/ASP-DOCA NPs were spherical, with an average particle size of 190–200 nm. The release of ORI from NPs was carried out in a pH-dependent manner, and ORI was released faster in acidic conditions (pH 5.0) than in physiological conditions (pH 7.4). Cell experiments demonstrated that ORI/ASP-DOCA NPs exhibited higher cell cytotoxicity and cellular uptake than free ORI. Moreover, ORI/ASP-DOCA NPs showed excellent ASGPR-mediated tumor targeting ability in H22 tumor-bearing mice and achieved a significant antitumor effect while reducing systemic toxicity. Therefore, these findings suggested that ASP-based NPs had potential for application as a new drug carrier for the liver-targeted delivery of ORI.

## Figures and Tables

**Table 1:** The particle size, PDI and zeta potential of ASP-DOCA NPs and ORI/ASP-DOCA NPs ( $\bar{X}$ ± SD, *n* = 3).

NPs	Particle Size (nm)	PDI	Zeta Potential (mV)
ASP-DOCA	187.6 ± 3.3	0.246 ± 0.015	−12.38 ± 4.72
ORI/ASP-DOCA	195.1 ± 3.4	0.233 ± 0.018	−18.96 ± 5.14

## Data Availability

The data presented in this study are available in this article.
