# Correction: Sun et al. Angelica Sinensis Polysaccharide-Based Nanoparticles for Liver-Targeted Delivery of Oridonin. *Molecules* 2024, *29*, 731

**DOI:** 10.3390/molecules30224326

**Published:** 2025-11-07

**Authors:** Henglai Sun, Jijuan Nai, Biqi Deng, Zhen Zheng, Xuemei Chen, Chao Zhang, Huagang Sheng, Liqiao Zhu

**Affiliations:** 1 College of Pharmacy, Shandong University of Traditional Chinese Medicine, Jinan 250355, China; 2 Key Laboratory of Traditional Chinese Medicine Classical Theory, Ministry of Education, Shandong University of Traditional Chinese Medicine, Jinan 250355, China

**Figure 11B,C Legend**

In the original publication, there was a mistake in the legend for Figure 11B,C. The experiment duration was incorrectly stated as 6 h instead of 4 h. The correct legend appears below.

(**B**) CLSM images of HepG2 cells incubated with different drugs for 4 h. (**C**) CLSM images of HeLa cells incubated with different drugs for 4 h.

**Error in Figure 12A,B**

In the original publication [1], there were mistakes in Figure 12A,B as published. The mouse images in Figure 12A for DIR/DEX-DOCA at 2 h and 12 h and for DIR at 4 h, 6 h, and 12 h were incorrect. In addition, Figure 12B contained a duplication error for DIR. The corrected Figure 12A,B are shown below.

The authors state that the scientific conclusions are unaffected. This correction was approved by the academic editor. The original publication has also been updated.

## Figures and Tables

**Figure 12:**
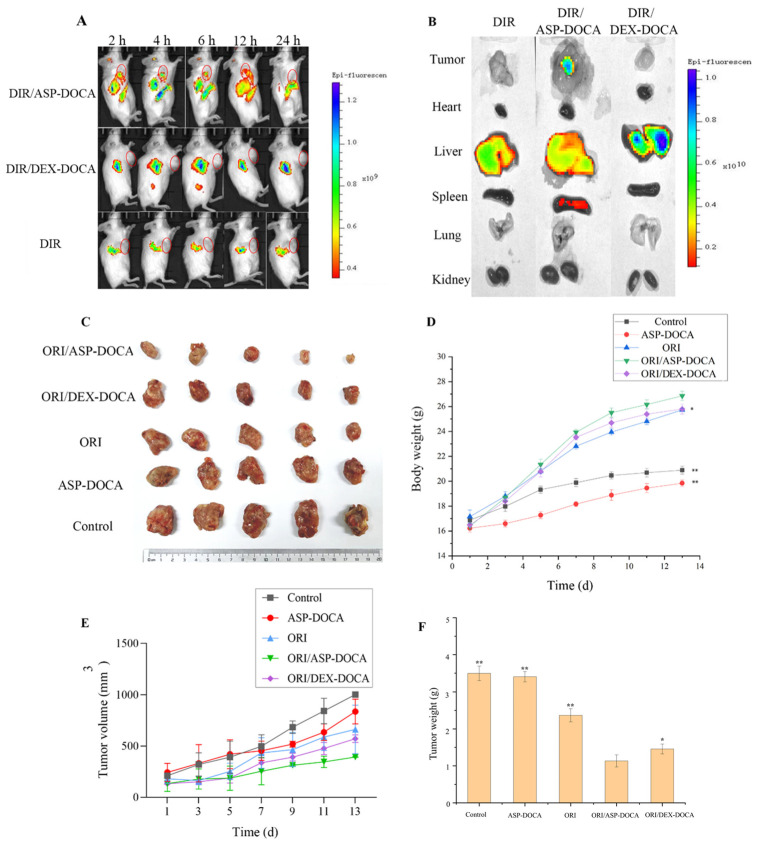
(**A**) Fluorescence images of DIR obtained by free DIR, DIR/ASP-DOCA and DIR/DEX-DOCA NPs at different time points of tumor-bearing mice in vivo; (**B**) DIR fluorescence imaging of the main isolated organs of tumor-bearing mice; (**C**) Tumor tissue images of tumor-bearing mice in each group; (**D**) Changes in the body weight of tumor-bearing mice in each group; (Note: compared with the ORI/ASP-DOCA NPs group, * *p* < 0.05, ** *p* < 0.01); (**E**) Changes in the tumor volume of tumor-bearing mice in each group; (**F**) Tumor tissue weight of tumor-bearing mice in each group (Note: compared with control group, * *p* < 0.05, ** *p* < 0.01).
